# Is hepatitis B birth dose vaccine needed in Africa?

**DOI:** 10.11604/pamj.supp.2017.27.3.11546

**Published:** 2017-06-22

**Authors:** Cynthia Raissa Tamandjou, Tongai Gibson Maponga, Nafiisah Chotun, Wolfgang Preiser, Monique Ingrid Andersson

**Affiliations:** 1 Division of Medical Virology, Faculty of Medicine and Health Sciences, Stellenbosch University, Cape Town, South Africa; 2 National Health Laboratory Service (NHLS), Tygerberg Hospital, Tygerberg, South Africa; 3 Oxford University Hospitals NHS Foundation Trust, Oxford, United Kingdom

**Keywords:** HBV, birth-dose, elimination, Africa

## Abstract

This commentary describes the need for a birth dose monovalent hepatitis B virus (HBV) vaccine and an effective programme for the prevention of mother-to-child-transmission (MTCT) of HBV in Africa. Current World Health Organization guidelines recommend routine maternal screening for HBV followed by treatment of highly infectious HBV-infected mothers, and HBV birth dose vaccination and the administration of hepatitis B immunoglobulin for HBV-exposed infants as an effective strategy for the prevention of HBV MTCT. None of these practices are currently in place in most parts of Africa. To date, fewer than 10 African countries vaccinate children at birth against HBV. Despite the hurdles associated with implementing this practice, its expansion to the rest of Africa is feasible and crucial to reducing the global number of new HBV infections by 90% by 2030, as targeted by the current Global Health Strategy for the elimination of viral hepatitis.

## Commentary

Hepatitis B virus (HBV) infection remains an important co-morbidity in the global HIV pandemic. The 2013 Global Disease Burden study showed that the total number of yearly deaths attributable to the complications of this infection was estimated at 686,000 and the number of life years lost was higher than for HIV [1]. Whilst extensive efforts and resources are invested in the control of HIV, HBV disease control lags far behind.

After East Asia, sub-Saharan Africa (SSA) carries the highest proportion of chronic hepatitis B (CHB) carriers, the majority of whom are unaware of their HBV status. The long-term sequelae of CHB infection include liver cirrhosis and hepatocellular carcinoma. The latter is reported as the third most common cause of cancer deaths worldwide in the 2012 GLOBOCAN report [2].

CHB is usually a consequence of acquisition of the virus during early childhood. The proportion of infected individuals who develop CHB decreases with age: from about 90% of children infected perinatally, to 30%-50% of those infected as toddlers to less than 5% in adulthood [3]. Data from The Gambia suggest that the majority of chronic carriers with severe liver fibrosis and those requiring treatment may be attributable to HBV infections acquired through mother-to-child transmission (MTCT) [4]. Therefore, preventing MTCT of HBV is crucial to breaking the transmission cycle of the virus and preventing the expansion of the current pool of infected individuals.

**Figure 1:**
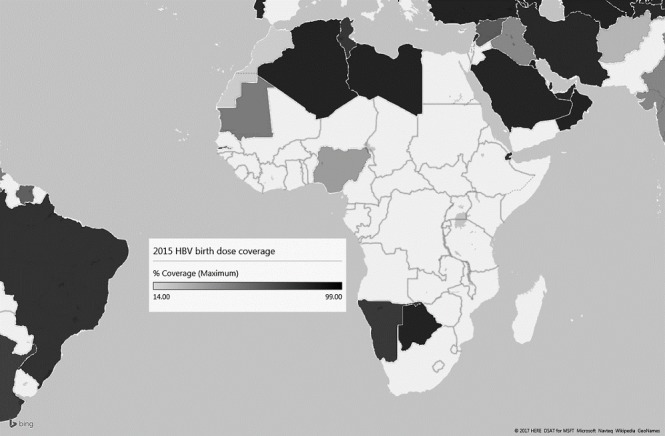
**Map of HBV birth dose vaccine and in-country coverage rates for 2015.** Only nine African countries provide HBV birth dose vaccine and where provided, coverage needs to improve in order to achieve the recommended targets set by the WHO.

Elimination of HBV is achievable more quickly if we can step up efforts to prevent HBV MTCT. In May 2016, the World Health Assembly announced the first ever Global Health Strategy for the elimination of viral hepatitis as a public health threat globally by reducing mortality due to viral hepatitis by 65% and the number of new infections by 90% by 2030. To reduce new infections, the strategy seeks to increase the global coverage of the hepatitis B birth dose vaccine, from the current 38% to 90%. World Health Organization (WHO)/United Nations International Children´s Fund (UNICEF) coverage estimates for 2015 suggest that HBV birth dose vaccine coverage in Africa is the lowest globally, at only 10% [5]. As shown in Figure 1, fewer than 10 countries in SSA offered the HBV birth dose vaccine in 2015, and even among these countries, coverage rates remain poor. A great proportion of SSA countries only offer the HBV vaccination from 6 weeks of age and thereafter at 10 and 14 weeks, as a multivalent vaccine formulation. Multivalent paediatric vaccine formulations offer the benefits of low cost and increased vaccine coverage [6]. However, the introduction of multivalent vaccines that contain hepatitis B surface antigen (HBsAg) into African EPIs will make implementation of the HBV birth dose vaccine more difficult. In South Africa for example, the pentavalent vaccine (DTaP-Hib-IPV) has been phased out and replaced by the hexavalent vaccine, with the addition of hepatitis B (DTaP-Hib-IPV-HepB), which cannot be administered at birth. The monovalent HBV vaccine that was previously used as part of the EPI is no longer available for administration at birth to HBV-exposed infants.

Despite improved immunization coverage rates achieved through combination vaccines, the risk of vertically-transmitted HBV infection is significant for infants who are born to highly-infectious HBV-infected mothers. Infants remain vulnerable to infection from vertically transmitted virus during the six weeks before they receive the first dose of the HBV vaccine. Vaccination at six weeks will reduce the risk of horizontal transmission but may be too late to halt the cycle of infection from mother to child.

Current HBV vaccination schedules in Africa are based on data generated from early HBV studies, where horizontal transmission was identified as the predominant route of transmission of this virus, causing around 90% of chronic infections while the remaining 10% was attributed to MTCT. Rates of vertical transmission were considered low because of the low prevalence of hepatitis B e antigen (HBeAg) among African pregnant women and therefore lower hepatitis B viral loads and risk of vertical transmission. However, data from Africa have emerged showing that the prevalence of HBeAg among HBV-infected pregnant women may not be as low as previously thought, with around 10% of all HBV-infected pregnant women being HBeAg seropositive, regardless of HIV status [7]. In the case of co-infection with HIV, the risk of transmission may increase by 2.5-fold when compared to the risk of transmission from HBV mono-infected mothers [8]. In a recent study in Malawi, 10% (5/51) of children born to HIV/HBV co-infected women became HBV-infected, despite vaccination administered at six weeks [9]. A similar HBV transmission rate from HIV/HBV co-infected women was reported in South Africa despite vaccination at 6, 10, and 14 weeks as per the South African EPI schedule [10]. Furthermore, a recent meta-analysis revealed that the annual number of HBV perinatal infections in SSA (n = 367 250) is estimated to be twice the number of HIV perinatal infections (n = 190 000) [11], indicating that identifying women at risk of transmitting the infection to their infants is crucial to prevent HBV MTCT.

Despite these troubling data and current WHO guidelines strongly recommending antenatal routine screening in countries with a HBsAg seroprevalence ≥2% in the general population [12], most African countries do not offer maternal screening for HBV infection - in part due to a lack of resources. While it is true that laboratory testing is expensive and often limited to certain parts of SSA and certain laboratories, advances in technology have allowed the development of simple rapid diagnostic tests (RDTs). Simple rapid immunochromatographic tests for HBsAg screening are widely available commercially. These tests generally have an acceptable diagnostic sensitivity and specificity, are reliable and robust, and offer advantages such as low cost and portability thereby allowing testing at the point-of-care level and decreasing loss to follow-up of patients. Therefore, rapid-test based screening for HBsAg of pregnant women is an important tool for the prompt identification of those women that are HBV-infected.

HBV MTCT prevention services can be incorporated into the existing and extensive HIV MTCT prevention infrastructure that is already in place in most of SSA, thus reducing costs of introducing a new screening test. Combination rapid tests that are able to detect HIV, syphilis, and HBsAg in pregnant women are desirable for the integration of MTCT prevention services. Also appealing are combination rapid tests that are able to detect both HBsAg and HBeAg, which is a marker of high infectivity and active viral replication. Therefore, these tests would be able to identify those women at high risk of transmitting HBV to their infants. Affordable, Sensitive, Specific, User-friendly, Rapid and Robust, Equipment-free, and Deliverable to end-users (ASSURED) combination tests will present a significant step forward towards efforts to control maternally transmitted infections.

Identifying HBV-infected pregnant women early in their pregnancy would allow their linkage to care, where those women at high risk of transmission could access efficacious antiviral therapy. The use of antivirals such as tenofovir in the third trimester, together with vaccination of infants at the time of birth has been shown to prevent HBV MTCT [13, 14]. However, more needs to be done to improve access to these drugs at lower prices for those who are HBV mono-infected. Ideally, this strategy should be supplemented by the administration of hepatitis B immunoglobulin (HBIG) at birth. However, this is not feasible in SSA at present due to concerns associated with cost and supply of HBIG.

High-level political commitment is required in order to have functional and effective HBV MTCT prevention - a key component missing in much of SSA. This calls for a prevention strategy that includes bringing HBV vaccination closer to the time of birth. Although costs and the limited availability of resources may be important issues to consider, vaccinating children at birth is feasible and cost-effective in SSA [15, 16]. Large-scale screening using RDTs followed by treatment is also feasible and cost-effective [17, 18].

Implementation of the HBV monovalent birth dose vaccine has been impeded by a number of barriers, exhaustively detailed in the Practices to improve coverage of the hepatitis B birth dose vaccine document of the WHO [19]. Political willingness, services delivery arrangements, especially for those babies born outside of health facilities, and the cold chain issue appear as the main obstacles faced in the African setting. Planning and allocation of human and financial resources, which in turn is to a large extent determined by the political willingness to invest in prevention of HBV MTCT, are required. There is therefore an important part to be played by health advocacy organizations in bringing political attention to the burden of CHB infections. Implementation will also require dealing with the existing challenge of reaching infants who are born away from health facilities. An estimated 25% of urban births compared to 60% of rural births in SSA occur outside of health facilities [20]. These infants will need to be reached within 24 hours of birth either by taking the vaccine to the infant through community outreach or encouraging bringing the newborn to a health facility shortly after birth through education of the mothers during the antenatal period or support at the time of birth.

Data from Lao People´s Democratic Republic, a resource-limited country, shows that the HBV birth dose vaccine can indeed be stored outside the cold chain whilst maintaining stability for up to 28 days [21]. Moreover, the use of single-dose vials such as compact prefilled auto-disable devices is an innovative way of providing HBV birth dose vaccine that can be administered out of the health facility in the event of home births. These devices cannot be re-used and are equipped with a vaccine vial monitor that indicates when the vaccine vial has been exposed to excessive heat. Access to such innovative forms of the vaccine together with training of health care workers would improve HBV birth dose coverage in remote areas where cold chain facilities for vaccine storage may not be available. Community health workers are also important in encouraging HBV birth dose vaccination for children born out of health facilities.

Africa needs the HBV birth dose vaccine if we are to achieve the goal of eliminating HBV MTCT. Although there are still hurdles to overcome for its successful implementation, with concerted efforts from all stakeholders, it is achievable. Screening pregnant women for HBV and treating those with high viral loads would further reduce the risk of vertical transmission. The combined use of these practices would serve to effectively break the HBV transmission cycle, bringing us closer to eliminating HBV in Africa, with considerable medical and socioeconomic benefits.

## Competing interests

The authors declare no competing interests.
